# DNA damage in human glomerular endothelial cells induces nodular glomerulosclerosis via an ATR and ANXA2 pathway

**DOI:** 10.1038/s41598-020-79106-3

**Published:** 2020-12-17

**Authors:** Ai Fujii, Yumi Sunatani, Kengo Furuichi, Keiji Fujimoto, Hiroki Adachi, Kuniyoshi Iwabuchi, Hitoshi Yokoyama

**Affiliations:** 1 Department of Nephrology, School of Medicine, Kanazawa Medical University, 1-1 Daigaku, Uchinada, Ishikawa 920-0293 Japan; 2 Department of Biochemistry I, School of Medicine, Kanazawa Medical University, Uchinada, Japan

**Keywords:** Nephrology, Pathogenesis

## Abstract

Collagen type VI (COL6) deposition occurs in various glomerular diseases, causing serious pathological damage like nodular lesions. However, the mechanisms underlying the deposition of COL6 remain unclear. In renal biopsy samples, immunohistochemical analyses revealed that COL6 and phosphorylated histone H2AX (γ-H2AX), a DNA damage marker, were detected mainly in diabetic nodular glomerulosclerosis, in which the γ-H2AX-positive area was identified as the independent factor significantly associated with the COL6-positive area (β: 0.539, t = 2.668). In in vitro studies, COL6 secretion from human renal glomerular endothelial cells (HRGECs) was assessed by measuring the decrease in the cytoplasmic COL6-positive cells and an increase in the amount of COL6 in the culture medium. Mitomycin C (MMc) treatment of HRGECs increased the number of γ-H2AX-positive cells and COL6 secretion, which were suppressed by a specific inhibitor of ataxia telangiectasia and Rad3-related (ATR). MMc-induced COL6 secretion was also suppressed by Annexin A2 (ANXA2) siRNA transfection. Moreover, the inhibition of ATR activity did not induce any extra suppression in the MMc-induced COL6 secretion by ANXA2 siRNA transfected cells. These results confirm that nodular glomerulosclerosis partially results from DNA damage in the glomerulus and that DNA damage-induced COL6 secretion from HRGECs occurs through an ATR and ANXA2-mediated pathway.

## Introduction

Nodular glomerulosclerosis (Kimmelstiel-Wilson nodules) is one of the typical glomerular lesions in diabetic nephropathy. Diabetic nephropathy is the leading cause of end-stage kidney disease worldwide, and nodular glomerulosclerosis is a strong prognostic factor for kidney function^1^. Nodular glomerulosclerosis is not specific for diabetic nephropathy; it is also detected in many other kidney diseases derived from a number of causes, including smoking, obesity, and hypertension^2^. Collagen type VI (COL6) is one of the main components of nodular glomerulosclerosis^3^. The deposition of COL6 in nodular glomerulosclerosis in diabetic nephropathy has been extensively examined^3,4^. Of the 28 types of collagen, COL6 is the only collagen that assembles fibrous bodies in cells and is secreted by a mechanism involving exocytosis^5^. Accumulated COL6 in the extracellular matrix is typically resistant to collagenase^6^ and may have a negative impact on glomerular regeneration or repair. Therefore, the deposition of COL6 in nodular glomerulosclerosis might be a key mechanism in the progression of kidney disease. Thus, further studies that elucidate the mechanisms contributing to the deposition of COL6 in nodular glomerulosclerosis will be valuable for discovering new therapeutic targets for kidney disease. We previously demonstrated that DNA damage in the glomerular endothelial cells and COL6 deposition in the glomeruli are positively correlated in patients with renal grafts^7^. Both renal allografting and diabetic nephropathy induce DNA damage in glomerular endothelial cells^8,9^. DNA is continually damaged by external factors such as radiation, ultraviolet light, and various chemicals. Moreover, a mutation of FAN1, a nuclease involved in the repair of DNA damage triggered by DNA inter- and intra-crosslinking agents such as mitomycin C (MMc), has been reported to cause karyomegalic interstitial nephritis, a model disorder of renal fibrosis^10^, indicating the association between DNA damage and renal fibrosis.

Ataxia-telangiectasia mutated (ATM) and ataxia telangiectasia and Rad3-related (ATR) are the two main kinases that participate in DNA damage-induced checkpoint signaling^11^. ATM, together with its partner protein complex Mre11-Rad50-NBS1, senses DNA double-strand breaks (DSBs), which can be generated by DNA damaging agents such as ionizing radiation^12,13^, and radiation mimic anti-cancer drug Neocarzinostatin (NCS)^14^. ATR with ATR interacting protein, ATRIP, senses single-stranded DNA coated by a single-strand DNA binding protein, replication protein A. Single-stranded DNA is generated by stalling of replication forks and processing of various DNA damage including DSBs^11^.

Annexin A2 (ANXA2), a member of the calcium-dependent phospholipid-binding proteins, is predominantly located in the cytoplasm and translocates to the cell membrane upon elevation of cytoplasmic Ca^2+^^15^. Since a fraction of ANXA2 is located in the nucleus, ANXA2 has been considered to be a nuclear-cytoplasmic shuttling protein^16^. Its diverse functions include endocytosis, exocytosis, cell-matrix interactions, cell motility, signal transduction, transcription, mRNA transport, and DNA replication^17^. A recently performed study has revealed that ANXA2 participates in the secretion of COL6 by bronchial epithelial cells^18^.

In this study, we examined the relationship between DNA damage and COL6 deposition in human glomeruli with biopsy samples of various kidney diseases and investigated the mechanisms underlying COL6 secretion by DNA damage using cultured human renal glomerular endothelial cells (HRGECs).

## Results

### Characteristic of nodular glomerulosclerosis

A total of 180 patients was enrolled in this clinical study. Nodular glomerulosclerosis was detected in the samples of 14 (7.7%) of the 180 patients. The baseline clinical characteristics of patients with nodular lesions (N = 14) and disease controls (N = 16) at the time of renal biopsy are shown in Table 1. The mean systolic and diastolic blood pressures were 133.8 ± 18.2 and 75.9 ± 9.2 mmHg, respectively, in patients with nodular lesions, and 125.6 ± 19.8 and 66.9 ± 11.0 mmHg, respectively, in those without nodular lesions. No significant differences in mean systolic or diastolic blood pressures were observed between the two groups. Moreover, mean urinary protein excretion levels and serum creatinine (sCr) levels were 4.2 ± 4.3 g/gCr and 1.38 ± 0.5 mg/dL, respectively, in patients with nodular lesions, and 3.1 ± 3.6 g/gCr and 1.10 ± 0.7 mg/dL, respectively, in those without these lesions. No significant differences in urinary protein excretion levels and sCr levels were observed between the two groups. There were no significant differences in disease backgrounds between the two groups. In addition to these clinical data, no significant differences were noted in the data shown in Table 1.

**Table 1:** Baseline characteristics of subjects.

	All subjects	Nodular lesion (+)	Nodular lesion (−)	*p* values
	N = 30	N = 14	N = 16	(+) vs. (−)
Gender (male, %)	20 (66.7)	11 (78.6)	9 (56.2)	0.04
Age (years old)	54.3 ± 18.9	55.9 ± 14.8	52.9 ± 22.2	0.67
**Blood pressure (mmHg)**				
Systolic	129.4 ± 19.2	133.8 ± 18.2	125.6 ± 19.8	0.25
Diastolic	71.1 ± 11.0	75.9 ± 9.2	66.9 ± 11.0	0.22
Urinary protein (g/gCr)	3.6 ± 3.9	4.2 ± 4.2	3.1 ± 3.6	0.40
Serum creatinine (mg/dL)	1.22 ± 0.58	1.37 ± 0.48	1.10 ± 0.65	0.22
HbA1c (%)	6.1 ± 1.1	6.4 ± 1.4	5.8 ± 0.8	0.16
Diabetes mellitus	8	3	5	N.S.
Nephrosclerosis	6	3	3	
Transplanted allografts	11	6	5	
Minor glomerular abnormality	5	0	5	

The population included 30 patients with or without nodular lesions.

Values are means ± SD, N.S., not significant.

Comparisons between groups were evaluated by the Mann–Whitney U test.

### DNA damage is correlated with COL6 deposition and nodular glomerulosclerosis in diseased renal biopsy samples

**Figure 1:**
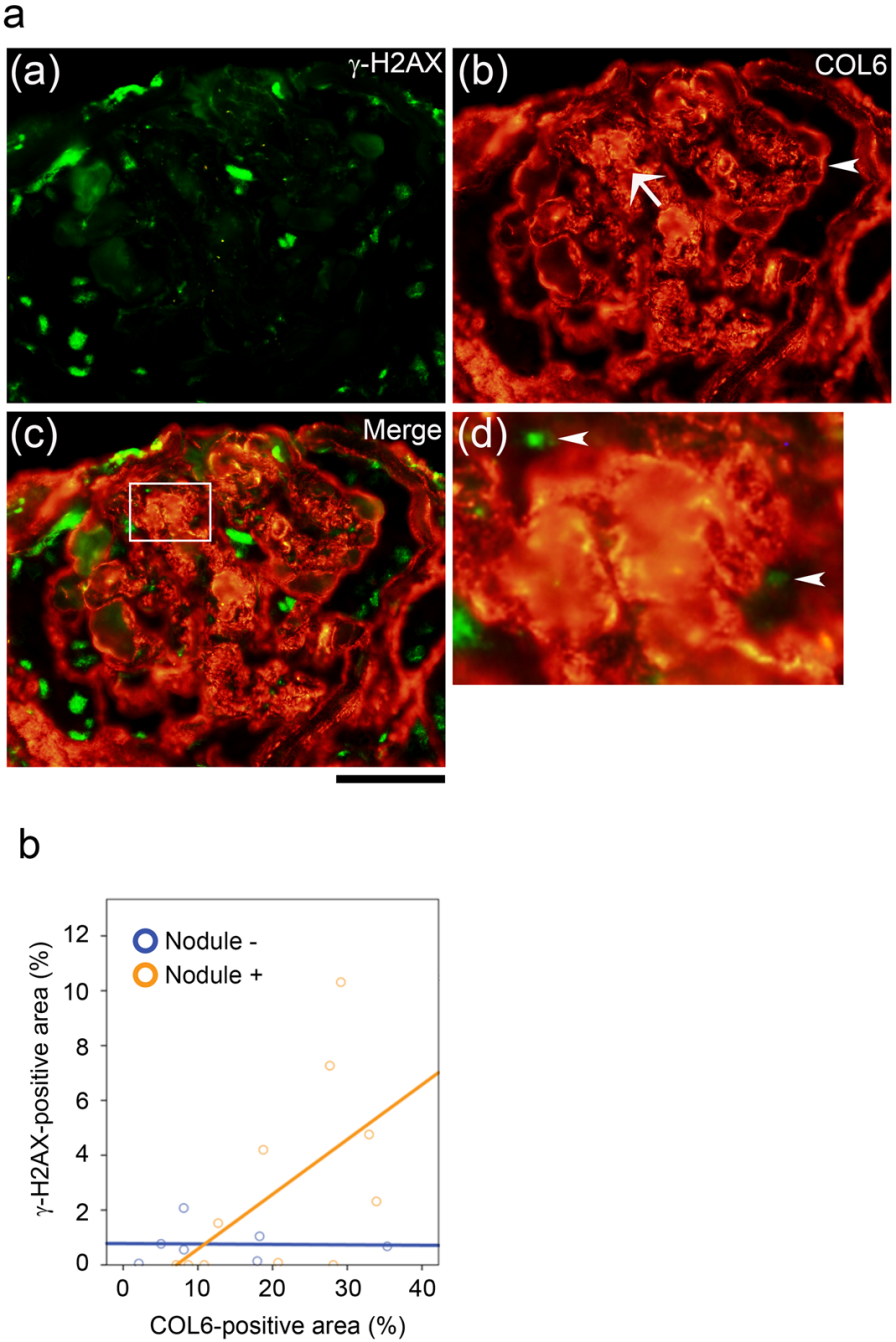
Characteristics of nodular glomerulosclerosis. [**a**] Double staining for γ-H2AX (**a**), COL6 (**b**), and both (**c**). Scale bar: 50 µm. (**d**) High power image of the white square of (**c**). Representative images of typical diabetic nodular glomerulosclerosis are shown. The arrow shows the nodular lesion, and the arrowhead shows a capillary loop in (**b**). The arrowheads in (**d**) show the γ-H2AX-positive nuclei of glomerular capillary cells. [**b**] The percentage of γ-H2AX-positive area and COL6-positive area was correlated in patients with nodular glomerulosclerosis (rS = 0.642, *p* = 0.017).

We first examined the relationship between DNA damage and glomerular fibrosis in this clinical study. Renal biopsy samples were subjected to immunohistochemical analyses to detect COL6 and γ-H2AX, phosphorylated Histone H2A, which is detected at sites of DNA damage, and a sensitive marker of DNA damage. Representative images of typical nodular glomerulosclerosis in diabetic nephropathy with hypertension are shown in Fig. 1a. γ-H2AX was detected in the nuclei of glomerular capillary cells, mainly endothelial cells, as previously reported^7^ (Fig. 1a). In addition, COL6 deposits were detected in capillary loops and nodular lesions (Fig. 1b–d). γ-H2AX and COL6 were examined in 25 samples (8 diabetes mellitus, 6 nephrosclerosis, and 11 transplanted allografts) and 5 samples of minimal change disease as controls. The baseline pathological characteristics of each group are shown in Table 2. Pathological scores and the percentage of the COL6-positive area were positively correlated with the presence of nodular lesions (rS = 0.571, *p* = 0.001), exudative lesions (rS = 0.435, *p* = 0.016), global glomerulosclerosis (rS = 0.387, *p* = 0.035), glomerular expansion (rS = 0.49, *p* = 0.006), and arteriolar hyalinosis (rS = 0.480, *p* = 0.007) upon Spearman’s test. The multiple regression analysis revealed that the percentage of the COL6-positive area is an independent factor associated both with the percentage of the γ-H2AX-positive area (β: 0.539, t = 2.668, *p* = 0.014) and the u-protein/creatinine ratio (uPCR) (β: − 0.494, t =  − 2.184, *p* = 0.039) (Table 3). Moreover, the percentage of the γ-H2AX-positive area and the COL6-positive area was significantly positively correlated in patients with nodular glomerulosclerosis (rS = 0.642, *p* = 0.017), but not in patients without it (Fig. 1b). These results suggest that DNA damage might be associated with the progression of nodular lesions caused by COL6 deposition in the glomerular capillary cells.

**Table 2:** Pathological findings in subgroups by glomerular nodular lesions.

Pathological findings of scores	Controls	Diabetes mellitus		*p* value	Nephrosclerosis		*p* value	Transplanted allograft		*p* value	Total		*p* value
	N = 5	N = 5	N = 3		N = 3	N = 3		N = 6	N = 5		N = 14	N = 16	
	( −)*	( +)*	( −)*		( +)*	( −)*		( +)*	( −)*		( +)*	( −)*	
**Glomerular lesions**													
Diffuse lesion (mesangial expansion)	0.00	3.00	1.33	0.42	1.00	1.33	0.01	1.83	0.80	0.07	1.94	0.87	< 0.01
Nodular lesion (nodular sclerosis)	0.00	1.00	0.00	< 0.01	1.00	0.00	< 0.01	1.00	0.00	< 0.01	1.00	0.00	< 0.01
Subendothelial space widening (double contour of the basement membrane)	0.00	2.60	1.67	0.40	0.67	2.00	0.49	1.83	0.20	0.04	1.7	0.97	< 0.01
Exudative lesion	0.00	1.00	0.67	0.42	0.33	0.67	0.52	0.67	0.20	0.15	0.67	0.38	< 0.01
Mesangiolysis microaneurysm	0.00	0.80	0.33	0.24	0.33	0.00	0.42	0.33	0.00	0.18	0.49	0.08	0.01
Peri-hilar neovascularization (polar vasculosis)	0.20	1.00	0.67	0.42	0.67	1.00	0.42	0.67	0.20	0.15	0.78	0.52	< 0.01
Global glomerulosclerosis Collapsing glomerulopathy Ischemic nephropathy	1.34	43.96	24.80	0.08	10.03	0.00	0.20	26.28	3.16	0.08	26.76	7.33	< 0.01
Segmental glomerulosclerosis	0.00	4.98	5.47	0.86	29.57	6.67	0.20	9.20	0.76	0.11	14.58	3.22	0.04
Glomerulomegaly	0.00	1.00	0.00	0.30	0.33	0.33	1.00	0.50	0.00	0.07	0.61	0.08	< 0.01
**Interstitial lesions**													
Interstitial fibrosis and tubular atrophy (IFTA)	0.80	2.60	2.00	0.88	1.67	0.67	0.67	2.50	1.20	0.03	2.26	1.17	< 0.01
Interstitial inflammation	1.00	2.60	2.67	0.42	1.67	0.67	0.67	2.50	1.20	0.03	2.26	1.38	< 0.01
Arteriolar hyalinosis	1.40	3.00	3.00	0.42	3.00	3.00	0.42	2.67	2.20	0.18	2.89	0.08	0.014
**Vascular lesions**													
Intimal thickening	1.40	2.00	1.67	0.26	2.00	1.67	0.59	2.00	1.60	0.38	2.00	1.58	0.09
Col6-positive rate	2.20	22.81	5.10	0.03	16.84	18.81	0.29	26.95	13.28	0.03	22.20	9.85	< 0.01
γ-H2AX-positive rate	1.94	2.87	0.46	0.16	1.40	1.01	0.71	3.22	0.80	0.22	2.50	1.05	0.11

Comparisons between groups were evaluated by the Mann–Whitney U test.

*Nodular lesions.

**Table 3:** Factors that influenced changes in Collagen type 6.

Objective variable: COL6-positive area	Standardization β	t	Significance**
γ-H2AX (%)	0.539	2.668	0.014
Average blood pressure (mmHg)	0.005	0.022	0.982
Diabetes mellitus (+) = 1, (−) = 0 *	−0.256	−1.273	0.216
uPCR (g/gCr)	−0.494	–2.184	0.039
sCr (mg/dL)	0.065	0.313	0.757
Age (year)	0.37	1.855	0.076

Abbreviations: COL6; Collagen type 6, γ-H2AX; Histone H2AX phosphorylation on Serine 139, sCr; serum creatinine levels. Mean blood pressure was calculated as (systolic blood pressure-diastolic blood pressure)/3 + diastolic blood pressure.

*Subjects with diabetes mellitus (DM) were classified as 1 and non-DM subjects as 0.

**Factors were analyzed by a linear multiple regression analysis.

### COL6 secretion and γ-H2AX appearance in cultured HRGECs after the MMc treatment

**Figure 2:**
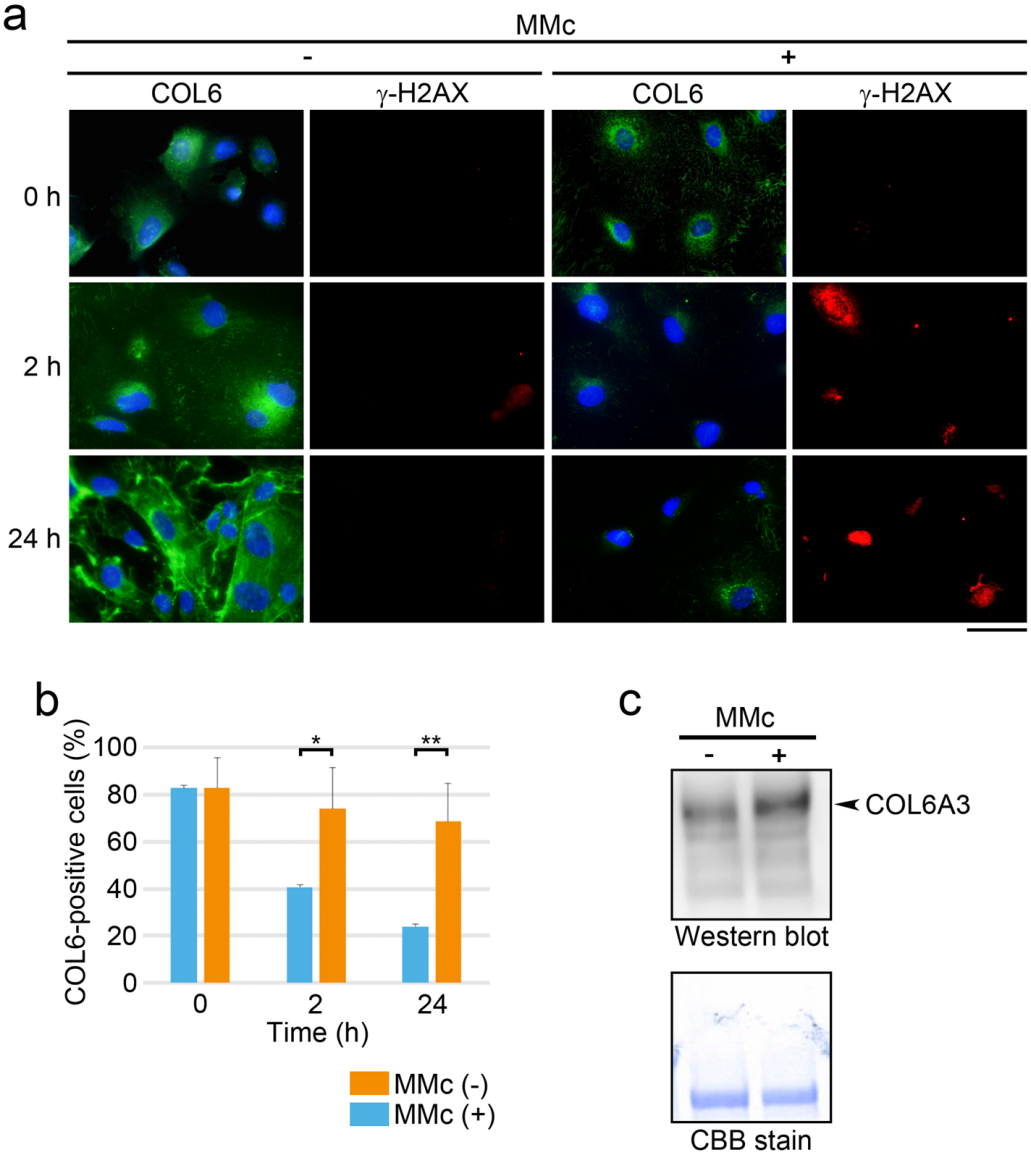
COL6 secretion after the MMc treatment. (**a**) HRGECs treated with (+) or without (−) MMc (12 µg/mL, 2 h) were washed out and cultured without MMc for the indicated number of hours. Cells were immunostained with anti-COL6 and anti- γ-H2AX antibodies. Scale bar: 50 µm. (**b**) The percentage of cells with cytoplasmic COL6 (COL6-positive cells) was measured using the samples of **a** by fluorescent images. A total of more than 100 cells of four independent fields per experiment was analyzed. Statistical significance was determined by the Mann–Whitney U test. *p < 0.05, **p < 0.01. (**c**) COL6 in the culture supernatants was detected by Western blotting. HRGECs were cultured with (+) or without (−) MMc (30 µg/mL) for 24 h. The lower panel showed CBB stain of the same membrane as a loading control.

To evaluate the DNA damage-induced COL6 secretion, primary-cultured HRGECs, potential COL6 secreting cells based on our previous study^7^, were treated with MMc. The survival rates were more than 90% after MMc treatment and were not significantly different from those of the non-treated cells (Supplementary Fig. S1). Immunofluorescence for COL6 and γ-H2AX in cultured HRGECs showed that COL6 was present in the cytoplasm with stronger signals around the nuclei of HRGECs before the MMc treatment, while γ-H2AX was not detected. After MMc treatment, γ-H2AX became positive at 2 h and 24 h, and the cytoplasmic COL6-positive cells decreased in a time-dependent manner (Fig. 2a,b). *COL6A3* mRNA was decreased after MMc treatment as determined by real-time reverse transcription-polymerase chain reaction (RT-PCR) (Supplementary Fig. S2), which may partially contribute to the decrease of the cytoplasmic COL6. Meanwhile, COL6 in the culture medium increased after MMc treatment (Fig. 2c). These data suggest a correlation between DNA damage and COL6 secretion in MMc-treated HRGECs.

### ATR participates in the secretion of COL6 by MMc-treated HRGECs

**Figure 3:**
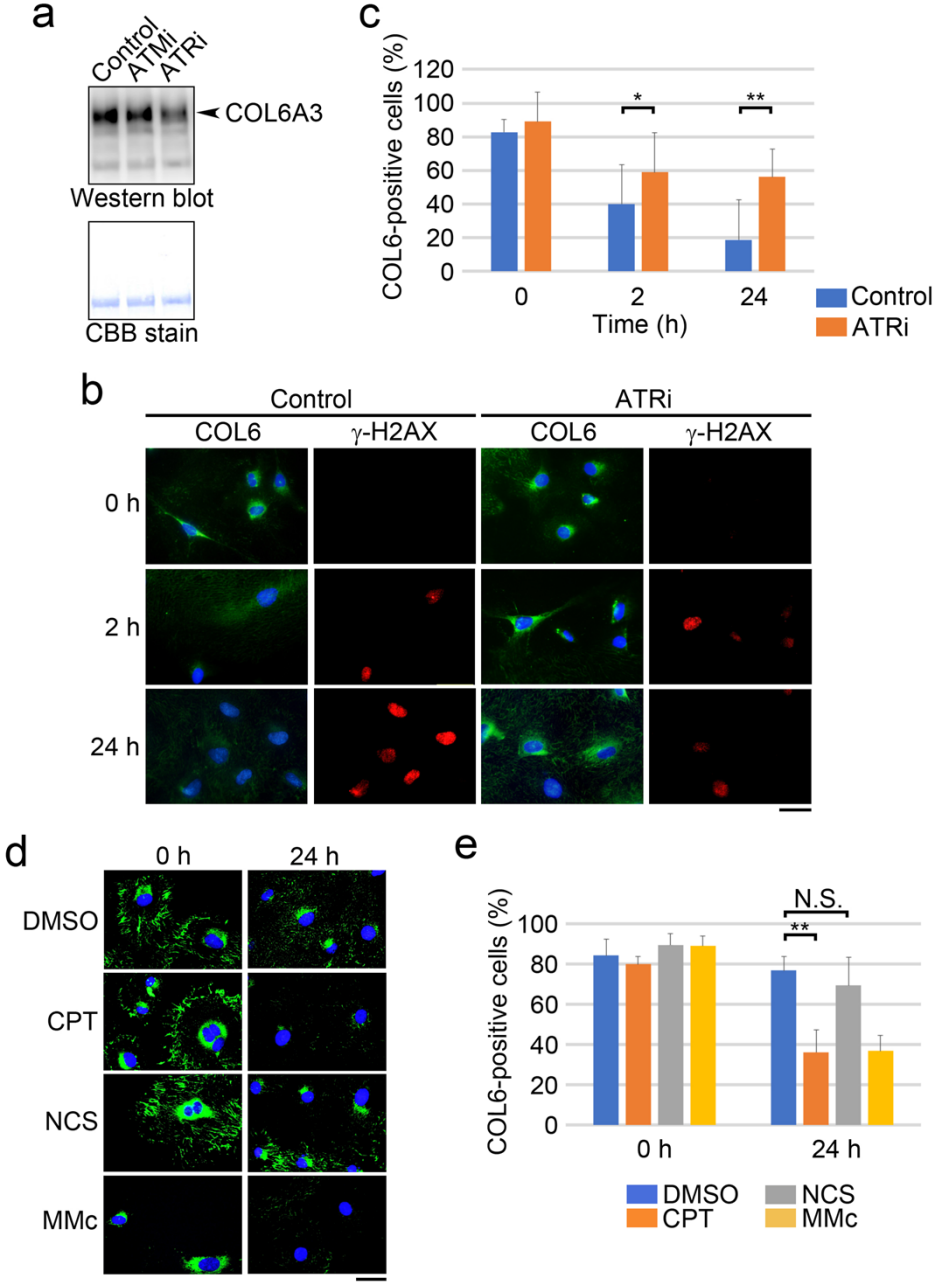
ATR is required for MMc-induced COL6 secretion. (**a**) COL6 in the culture supernatants was detected by Western blotting. HRGECs were cultured with DMSO (control), ATRi or ATMi for 1 h, then MMc (30 µg/mL) was added in the cultures. Twenty-four h after MMc addition, the supernatants were collected. The lower panel showed CBB stain of the same membrane as a loading control. (**b**) HRGECs were cultured with DMSO (control) or ATRi for 1 h, then MMc (12 µg/mL) was added in the cultures. Two h after MMc addition, the cells were washed and cultured with or without ATRi for the indicated number of hours. The cells were then immunostained with anti-COL6 and anti- γ-H2AX antibodies. Scale bar: 50 µm in each panel. (**c**) The percentage of cells with cytoplasmic COL6 (COL6-positive cells) was measured using the samples of **b** by fluorescent images. (**d**) HRGECs treated with DMSO, CPT (1.0 µM, 4 h), NCS (50 ng/mL, 4 h) or MMc (12 µg/mL, 2 h) were washed, and cultured without DNA-damaging agents for the indicated number of hours. Cells were immunostained with anti-COL6 antibody. Scale bar: 50 µm in each panel. (**e**) The percentage of cells with cytoplasmic COL6 (COL6-positive cells) was measured using the samples of **d** by fluorescent images. A total of more than 100 cells of four independent fields per experiment was analyzed. (**c**, **e**) Statistical significance was determined by the Mann–Whitney U test. *p < 0.05, **p < 0.01.

To determine which DNA damage signaling is activated after MMc treatment, we focused on ATR and ATM, which are major kinases that regulate DNA damage responses. Specific inhibitors for ATR or ATM were used to evaluate the contribution of ATR and/or ATM to MMc-induced COL6 secretion. MMc induced the phosphorylation of ATM target Chk2 and ATR target Chk1^19^. The ATM inhibitor (ATMi) suppressed the phosphorylation of Chk2, but not Chk1, and the ATR inhibitor (ATRi) showed the opposite effect (Supplementary Fig. S3). Western blot analysis with the culture medium revealed that the inhibition of ATR, but not ATM, suppressed MMc-induced COL6 secretion (Fig. 3a). Consistently, immunocytochemical staining revealed that the reduction in the cytoplasmic COL6-positive cells after MMc treatment was partially cancelled by the ATRi (control vs. ATR inhibited group, 2 h, 40 ± 24 vs. 59 ± 23, n = 5, *p* = 0.01; 24 h, 19 ± 24 vs. 56 ± 17, n = 5, *p* = 0.001) (Fig. 3b,c). In addition, the MMc-induced increase in the number of γ-H2AX positive cells was suppressed by ATR inhibition (Fig. 3b), suggesting the involvement of ATR in H2AX phosphorylation in MMc-treated HRGECs.

Next, we examined the effects of different DNA-damaging agents on COL6 secretion by HRGECs. NCS induces DSBs independent of DNA replication, and a topoisomerase I inhibitor, Camptothecin (CPT), induces DSBs in a replication dependent manner. Both CPT and NCS stimulated H2AX phosphorylation (Supplementary Fig. S4). However, only CPT elicited phosphorylation of Chk1 (Supplementary Fig. S5) and CPT, but not NCS, reduced the cytoplasmic COL6-positive cells (Fig. 3d,e). Collectively, these data suggest that ATR, but not ATM activity, is required for the secretion of COL6 by HRGECs after DNA damage.

### ANXA2 participates in the secretion of COL6 by MMc-treated HRGECs

**Figure 4:**
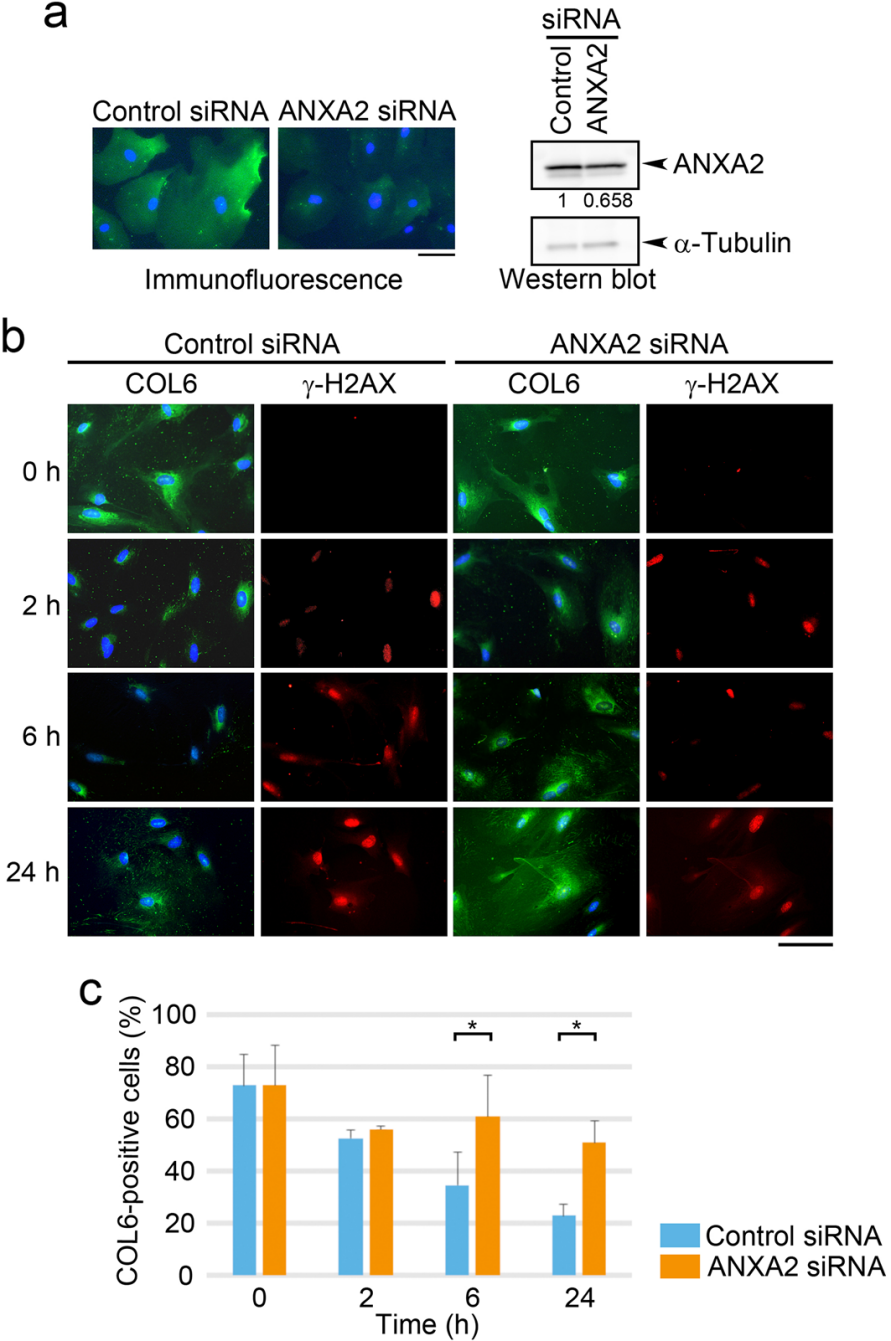
ANXA2 is required for MMc-induced COL6 secretion. (**a**) HRGECs were transfected with control or ANXA2 siRNA, and 48 h post-transfection, the protein levels of ANXA2 were measured by immunofluorescence (left panel) and Western blotting (right panel). The ratio of ANXA2 intensity of ANXA2 siRNA-treated cells versus control siRNA are shown below the ANXA2 blot. Data were normalized to the intensity of α-Tubulin loading control. (**b**) HRGECs transfected with control or ANXA2 siRNA were treated with MMc (12 µg/mL, 2 h). After washout, the cells were cultured without MMc for the indicated number of hours, and immunostained with anti-COL6 and anti- γ-H2AX antibodies. (**a**, **b**) Scale bar: 50 µm in each panel. (**c**) The percentage of cells with cytoplasmic COL6 (COL6-positive cells) was measured using the samples of **b** by fluorescent images. A total of more than 100 cells of four independent fields per experiment was analyzed. Statistical significance was determined by the Mann–Whitney U test. *p < 0.05.

A recent study has shown that ANXA2 plays a role in the protein transport of COL6 in bronchial epithelial cells^18^. Most of the ANXA2 is located in the cytoplasm but shuttles between the nucleus and cytoplasm in response to stress signals^17^. Thus, we examined the role of ANXA2 on DNA-damage-induced COL6 secretion in HRGECs. MMc treatment did not affect the amount of ANXA2 (Supplementary Fig. S6). ANXA2-siRNA transfection reduced ANXA2 expression as assessed by immunofluorescence analysis and western blot analysis (Fig. 4a). Again, the cytoplasmic COL6-positive cells were decreased, and γ-H2AX positive cells were increased after MMc treatment (Fig. 4b). However, this decline of the cytoplasmic COL6-positive cells were suppressed by ANXA2-siRNA transfection at 6 h (control vs. ANXA2 siRNA-transfected group, 35 ± 13 vs. 61 ± 16, n = 3, *p* = 0.02) and 24 h (23 ± 4 vs. 51 ± 8, n = 3, *p* = 0.012) (Fig. 4b,c). Similar results were obtained by the use of the two other ANXA2 siRNAs (Supplementary Figs. S7 and S8). The MMc-induced increase of the number of γ-H2AX positive cells was not affected by ANXA2-siRNA transfection. These results suggest that ANXA2 plays a role in secretion of COL6 in MMc-treated HRGECs.

### ATR might link to ANXA2 in MMc-induced COL6 secretion

**Figure 5:**
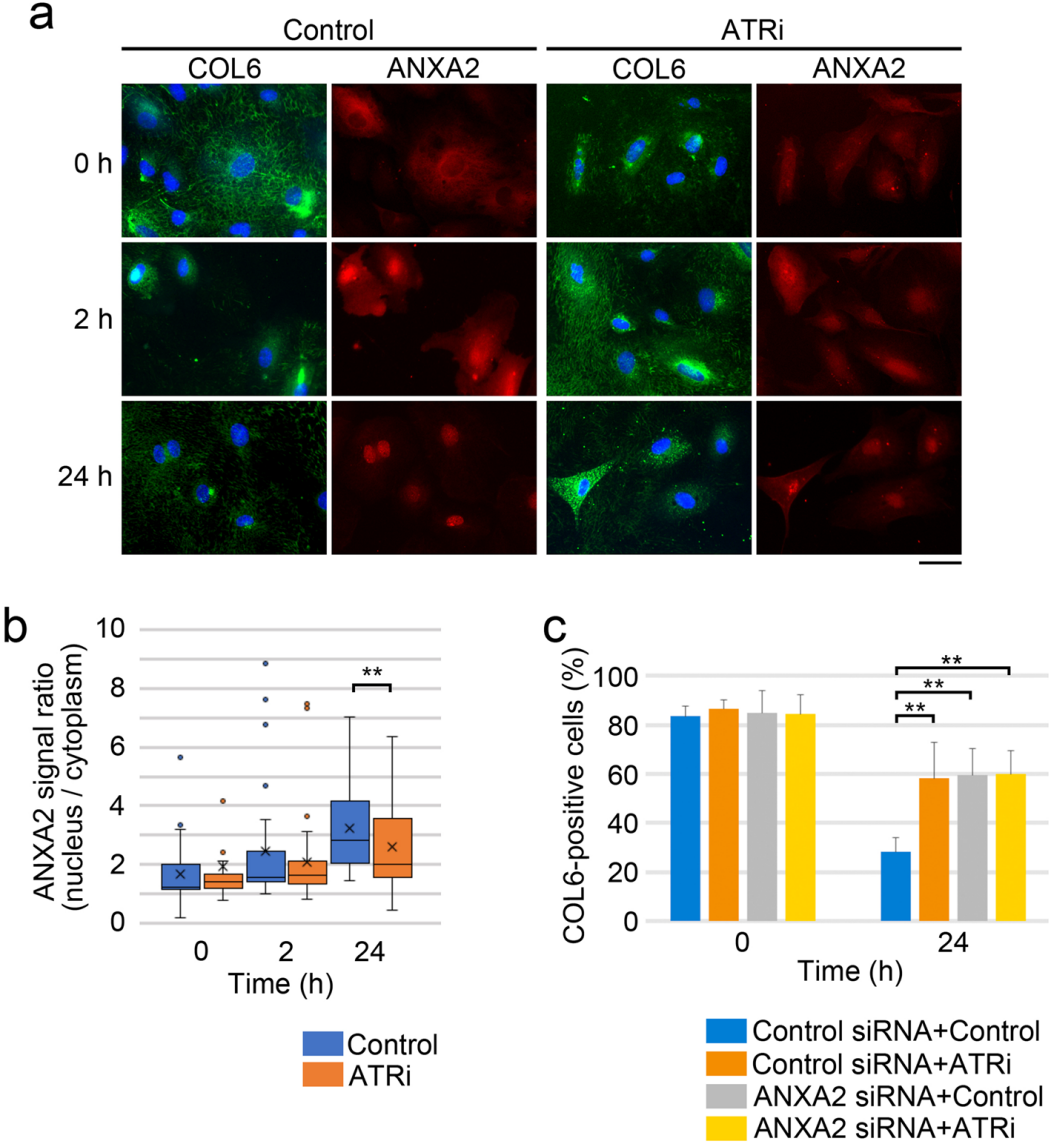
ATR may link to ANXA2 in MMc-induced COL6 secretion. (**a**) HRGECs were cultured with DMSO (control) or ATRi for 1 h, then MMc (12 µg/mL) was added in the cultures. Two h after MMc addition, the cells were washed and cultured with or without ATRi for the indicated number of hours. The cells were immunostained with anti-COL6 and anti-ANXA2 antibodies. Scale bar: 50 µm. (**b**) The ANXA2 signal ratio (nucleus/cytoplasm) were measured for more than 25 cells using the samples of **a**. (**c**) HRGECs transfected with control or ANXA2 siRNA were cultured with DMSO (control) or ATRi for 1 h, and then MMc (12 µg/mL) was added in the cultures. Two h after MMc addition, cells were washed, and cultured with or without ATRi for the indicated number of hours. The cells were immunostained with anti-COL6 antibody. The percentage of cells with cytoplasmic COL6 (COL6-positive cells) was measured by fluorescent images. A total of more than 100 cells of four independent fields per experiment was analyzed. (**b**, **c**) Statistical significance was determined by the Mann–Whitney U test. **p < 0.01.

ANXA2 accumulates in the nucleus in response to DNA damage^20^. Given that ATR and ANXA2 are required for DNA-damage-induced COL6 secretion, we investigated whether ATR activity affected the DNA-damage-induced nuclear accumulation of ANXA2. Immunofluorescence analyses revealed that the intensity of the ANXA2 signal in the nucleus increased after the MMc treatment (Fig. 5a). However, the ratio of its signal in the nucleus versus that in the cytoplasm was significantly lower in cells treated with the ATRi than that in control cells, suggesting that ATR activity enhances the DNA-damage-induced nuclear accumulation of ANXA2 (Fig. 5b). Finally, to determine functional interaction between ATR and ANXA2 in the MMc-induced COL6 secretion, the effect of combined ATR inhibition and ANXA2 suppression was examined. The single inhibition of ATR activity or ANXA2 expression comparably suppressed the MMc-induced decrease in the cytoplasmic COL6 positive cells, on which the combinatorial approach did not induce any additive effect (Fig. 5c). This result might link ATR with ANXA2-mediated COL6 secretion upon DNA damage.

## Discussion

In a previous study, we demonstrated that the presence of DNA damage and COL6 deposition are positively correlated in the glomerulus of renal graft patients^7^. In this study, we examined this correlation in various kidney diseases, especially focusing on those with nodular glomerulosclerosis. We found that DNA damage in the glomerulus is positively correlated with COL6 deposition and the formation of nodular glomerulosclerosis in various kidney diseases. Furthermore, we demonstrated that cultured HRGECs secrete COL6 upon DNA damage induced by MMc via an ATR- and ANXA2-mediated pathway.

Collagen is the main component of the extracellular matrix, and COL6 was previously shown to accumulate with nodular glomerulosclerosis in diabetic nephropathy^21^. COL6 has been reported to function as an early sensor of the injury/repair response and may regulate fibrogenesis by modulating cell-cell interactions^22^. However, once deposited, COL6 is completely resistant to metalloproteinases and is only digested by serine proteases secreted from neutrophils or mast cells^23^. Furthermore, COL6 stimulates the proliferation of mesenchymal cells and protects mesangial cells from apoptosis^24^. In this study, we demonstrate that DNA damage is correlated with COL6 deposition in the glomerulus. Thus, in addition to the resistance of COL6 to various proteases, DNA damage may be an important factor in the accumulation of COL6 in the glomerulus. Hypertension and hyperglycemia have been reported to induce ischemia and/or oxidative stress on glomerular endothelial cells^25^. These stresses may also cause DNA damage in endothelial cells. In this notion, diabetes and some toxic events of glomerular endothelial cells caused by monocrotaline injection were associated with the formation of nodular-like lesions in the obese-diabetic Otsuka Long-Evans Tokushima Fatty (OLETF) rat model^24^. Otherwise, it has been reported that COL6A3 levels are increased in tumors in response to exposure of cisplatin, another DNA crosslinking agent. DNA crosslinking agents may be one of inducers of COL6 accumulation in vivo^26^. Therefore, DNA damage detected by γ-H2AX in the glomerulus is a good prognostic marker for kidney diseases, particularly those with nodular glomerulosclerosis.

We demonstrated that ATR and ANXA2 are required for DNA-damage-induced COL6 secretion from HRGECs. ANXA2 accumulates in the nucleus upon DNA damage induced by various DNA damaging agents, such as ionizing radiation, ultraviolet radiation, etoposide, and chromium VI^20^. Although several studies have reported that ANXA2-phosphorylation regulates its cellular localization^27,28^, the association of DNA damage and ANXA2 phosphorylation is unclear. We, for the first time, showed the regulatory role of ATR for the DNA damage-induced nuclear accumulation of ANXA2. ANXA2 contains a nuclear export signal (NES), but does not contain a nuclear import signal, suggesting that ATR-dependent phosphorylation events inhibit the NES-mediated cytoplasmic export of ANXA2. However, ANXA2 does not contain the SQ/TQ motif (serine or threonine followed by glutamine). The serine or threonine residue of the SQ/TQ motif is a preferential target of ATR phosphorylation^29^. Thus, we speculate that certain proteins that mediate ANXA2 cytoplasmic exports via its NES are phosphorylated by ATR or its downstream kinases, such as Chk1, and thereby inhibiting the NES function of ANXA2.

How does ATR activity stimulate MMc-induced COL6 secretion? In addition to phosphorylation, ANXA2 has been reported to be subjected to various post-translational modification processes, such as acetylation, sumoylation, and ubiquitination^17^. ATR-dependent nuclear localization of ANXA2 may be necessary for such ANXA2 modification, and the modified ANXA2, after translocating to the cytoplasm, may stimulate the COL6 secretion. However, it is possible that the ATR-mediated ANXA2 nuclear retention and COL6 secretion are independent events. In bronchial epithelial cells, ANXA2 participates in COL6 secretion, associating at secretary vesicle membranes with COL6 and soluble N-ethylmaleimide-sensitive factor acceptor proteins 23, and vesicle-associated membrane protein 2 (VAMP2), members of the soluble N-ethylmaleimide-sensitive factor attachment protein receptor (SNARE) family of membrane fusion proteins^18^. Furthermore, in neurons, a fraction of ATR has been reported to be present in the cytoplasm, associated with synapsin-I and VAMP2^30^. Thus, an alternative possibility is that cytoplasmic ATR or its downstream kinases might phosphorylate components of the SNARE family of proteins, thereby stimulating COL6 secretion.

There are some limitations to this present study.

1. Difficulties were encountered when identifying the factors affecting γ-H2AX in a multivariate analysis.
2. This was a single-center study, and the number of cases examined was small. Therefore, a large-scale multicenter study will be required in the future.
3. In the present study, we did not stain SNARE proteins and were unable to compare COL6 deposition in vivo and in vitro.

In conclusion, we have herein demonstrated the positive correlation of COL6 deposition after DNA damage with nodular glomerulosclerosis in a clinical study. We have also demonstrated the novel mechanism of the COL6 secretion from HRGECs through an ATR and ANXA2 pathway, which is activated after DNA damage in vitro. These results provide novel insights into the development of new therapies that will suppress glomerulosclerosis in various kidney diseases.

## Methods

### Biopsy sample collection and pathological evaluation

A total of 180 renal biopsy samples was pathologically evaluated in this study. Kidney biopsies were performed between 2016 and 2019 in the Kanazawa Medical University. Among 180 renal biopsy samples, nodular glomerulosclerosis was detected in the glomeruli of 14 samples (7.7%); 5 diabetes mellitus, 3 nephrosclerosis, and 6 transplanted allografts. Sixteen samples without nodular glomerulosclerosis were collected as disease controls; 3 diabetes mellitus, 3 nephrosclerosis, 5 transplanted allografts, and 5 minimal change disease. All biopsy samples were evaluated under a light microscope after staining with periodic acid-Schiff, periodic acid methenamine silver, hematoxylin–eosin, and Masson’s trichrome stains, and were also examined by immunofluorescence and electron microscopy. Each pathological finding, including nodular glomerulosclerosis, was defined and scored according to a previous study (Supplementary Table)^1^. In brief, nodular glomerulosclerosis was defined as a rounded mesangial matrix expansion with no normal capillary being present around the expanded mesangial matrix. Clinical data at the time of renal biopsy were obtained from clinical records. The data we collected included urinary protein (g/gCr), the amount of occult urinary blood, sCr levels, and the estimated glomerular filtration rate (eGFR). The local Ethical Committee of the Kanazawa Medical University approved this study (Approval No.42). Written informed consent was obtained from all the participants before renal biopsy. The present study was conducted in accordance with the Declaration of Helsinki.

### Immunohistological analysis of subjects

γ-H2AX and COL6 were detected in diseased kidneys by immunohistochemistry. Fresh-frozen specimens were fixed by acetone before staining for γ-H2AX and COL6. We used 10% goat serum to block protein. A mouse monoclonal anti-human phospho-histone H2AX (Ser139) antibody (1000 µg/mL, 05-636, Merck Millipore, Burlington, MA) at a dilution of 1:200 and rabbit polyclonal anti-human COL6 antibody (250 µg/mL, AB7821, Sigma-aldrich, St. Louis, MO) at a dilution of 1:400 were used as the primary antibodies to detect γ-H2AX and COL6, respectively. The primary antibodies were incubated for 4 min in a microwave oven. Specimens incubated with each primary antibody were washed with phosphate-buffered saline (PBS) and stained using secondary antibodies: a goat anti-mouse IgG antibody labeled with Alexa Fluor 488 (A32723, Invitrogen, Carlsbad, CA) and a goat anti-rabbit IgG antibody labeled with Alexa Fluor 555 (A32732, Invitrogen) at a dilution of 1:400. Specimens stained by each secondary antibody were evaluated using the fluorescent microscope BZ-51 (Olympus, Tokyo, Japan). The percentage of COL6- and γ-H2AX-positive areas in each specimen was calculated using the Win ROOF 2015 software (Mitani Corporation, Fukui, Japan).

### Primary cell culture

HRGECs, which were described in a previous study^7^, were obtained from ScienCell Research Laboratories (PS-4000, San Diego, CA) and cultured following the manufacturer’s instructions. Between 2 and 5 cell passages were used in these experiments. To evaluate COL6 secretion in DNA-damaged HRGECs, COL6 levels in cells and the culture medium were measured. Sub-confluent cultured HRGECs were cultured with MMc (12 or 30 µg/mL, 20898-21, Nakarai tesque, Kyoto, Japan), NCS (50 ng/mL, N9162, Sigma-aldrich) or CPT (1.0 µM, C9911, Sigma-aldrich). VE-821 (SML1415, Sigma-aldrich, or 1893, Axon Medchem, Groningen, Netherlands) was used as an ATR inhibitor (ATRi) and KU55933 (A10506, AdoorQ BioScience, Irvine, CA) as an ATM inhibitor (ATMi) at a final concentration of 10 µM.

### Immunofluorescence

Fluorescence cell imaging procedures were performed as follows^7^. Cells were fixed with 4% paraformaldehyde at room temperature for 20 min, and were permeabilized with 0.1% Triton X-100/10% FBS/PBS for 1 h. To detect ANXA2, γ-H2AX, or COL6, cells were stained using a monoclonal mouse anti-human ANXA2 antibody (200 µg/mL, sc-28385, Santa Cruz Biotechnology, Dallas, TX) at a dilution of 1:50, a mouse monoclonal anti-human phospho-histone H2AX (Ser139) antibody at a dilution of 1:250, and a polyclonal rabbit anti-human COL6 antibody at a dilution of 1:2500, respectively. After staining with each primary antibody, cells were stained using the following secondary antibodies: the goat anti-mouse IgG labeled with Alexa Fluor 594 and goat anti-rabbit IgG labeled with Alexa Fluor 488 at a dilution of 1:1000. Nuclei were stained with 6-diamidino-2-phenylindole (DAPI) (P36934, Invitrogen). Fluorescence images were detected under fluorescent microscopes (BZX700, Keyence, Osaka, Japan, BX-51, Olympus, or IX71, Olympus). The number of cells that were positive for γ-H2AX in the nucleus or COL6 in the cytoplasm was determined using Win ROOF 2015 software. The signal intensity of ANXA2 (Fig. 5b) was measured by Image J^31^.

### Western blotting

Cells were lysed in Laemmli buffer with 1% protease inhibitor cocktail (P8340, Sigma-aldrich). Protein concentrations were estimated using the bicinchoninic acid assay (BCA) (23227, Thermo Fisher, Waltham, MA). Proteins were separated by SDS-PAGE using a 5%-20% gradient gel and transferred onto 0.45 µm PVDF blotting membranes (IPVH00010, Merck Millipore). The subsequent procedures were described earlier on^7^. After chemiluminescent detection, the membranes were stained with Coomassie brilliant blue (CBB) and scanned. The primary antibodies used in this study were as follows: anti- γ H2AX, anti-ANXA2, and anti-α-Tubulin (66031-1-Ig, Proteintech, Rosemont, IL). α-Tubulin was used as a loading control. The densitometry of ANXA2 and α-Tubulin was measured using Image J. Uncropped images of all Western blots are shown at Supplementary Fig. S9.

### siRNA treatment

siRNAs specifically targeting human *ANXA2* mRNA (SASI_Hs01_00246294, Sigma-aldrich) or a siRNA control (SIC002, Sigma-aldrich) at a final concentration of 20 µM were transfected into sub-confluent HRGECs using Lipofectamine RNAiMAX transfection reagent (13778, Invitrogen) in accordance with the manufacturer’s instructions. Forty-eight h after transfection, the medium was replaced with a normal medium.

### Statistical analysis

Descriptive statistics are presented as means ± standard deviation (SD) or medians (interquartile range). Comparisons between groups were performed by the Mann–Whitney U test. Clinicopathological data were analyzed using the Spearman’s method to compare COL6 deposition in kidney samples of minimal change disease, diabetes mellitus, nephrosclerosis, and transplanted allografts. The factors influencing COL6 deposition and nodular glomerulosclerosis were analyzed using multiple regression analysis. All analyses were performed using Statistical Package for Social Science ver. 16.0 (SPSS, Tokyo, Japan)*.* Values of *p* less than 0.05 were regarded as statistically significant.

## Supplementary Information

Supplementary Information.
