# Supplementary material for: DNA damage in human glomerular endothelial cells induces nodular glomerulosclerosis via an ATR and ANXA2 pathway
Source: Sci Rep. 2020 Dec 17;10:22206. doi: 10.1038/s41598-020-79106-3 (PMC7747722; doi:10.1038/s41598-020-79106-3)
Supplement: Supplementary file 1 — Supplementary Information. [file 41598_2020_79106_MOESM1_ESM.pdf]

DNA damage in human glomerular endothelial cells induces nodular glomerulosclerosis via an ATR and ANXA2 pathway

Ai Fujii[1]†, Yumi Sunatani[2]†, Kengo Furuichi[1], Keiji Fujimoto[1], Hiroki Adachi[1], Kuniyoshi Iwabuchi[2] & Hitoshi Yokoyama[1]*

Department of Nephrology[1] and Department of Biochemistry I[2], School of Medicine, Kanazawa Medical University

†:Ai Fujii and Yumi Sunatani both contributed equally to this manuscript.

*Corresponding Author

Hitoshi Yokoyama, MD, PhD

Department of Nephrology, School of Medicine, Kanazawa Medical University

1-1 Daigaku, Uchinada, Ishikawa 920-0293, Japan

E-mail: h-yoko@kanazawa-med.ac.jp

Tel: +81-76-218-8166

Fax: +81-76-286-2786

Supplementary Figure S1

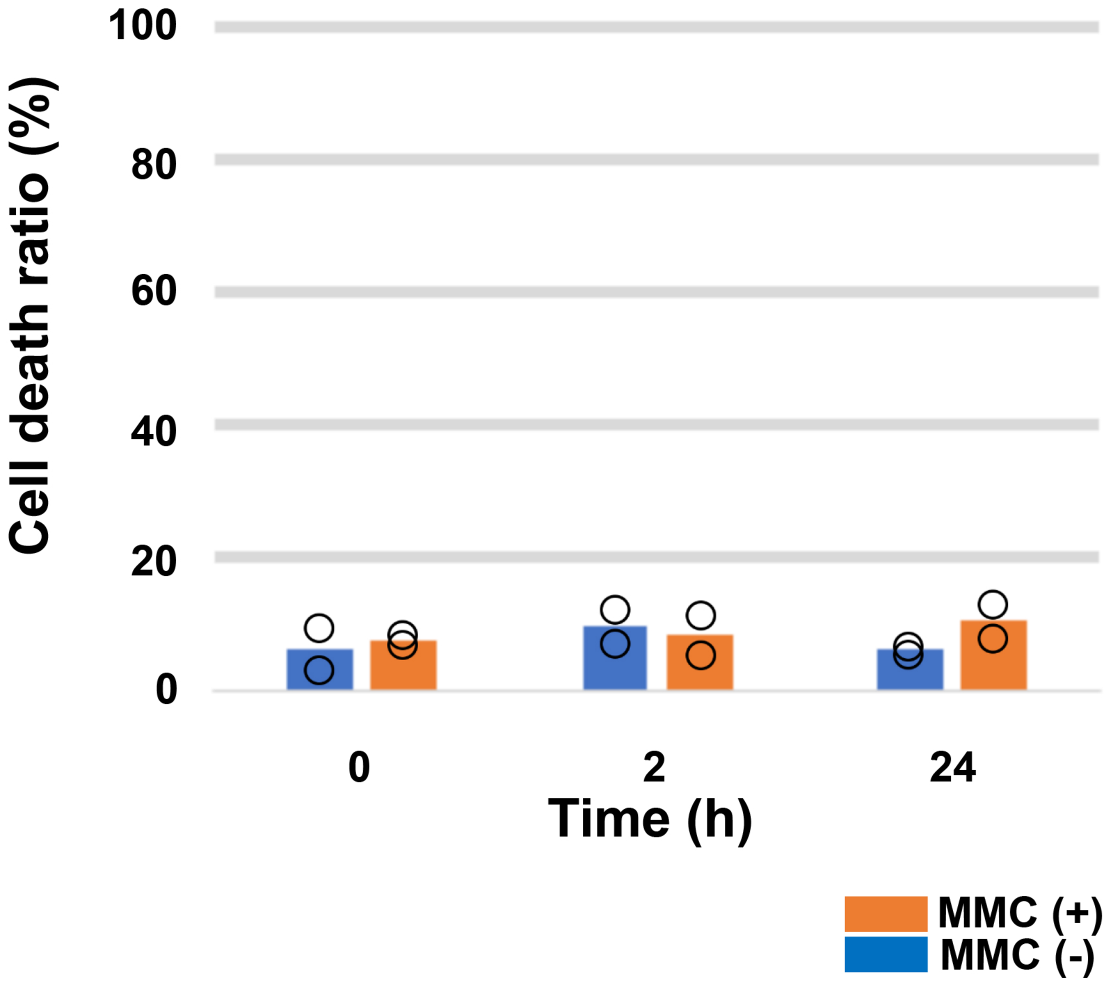

Supplementary Figure S2

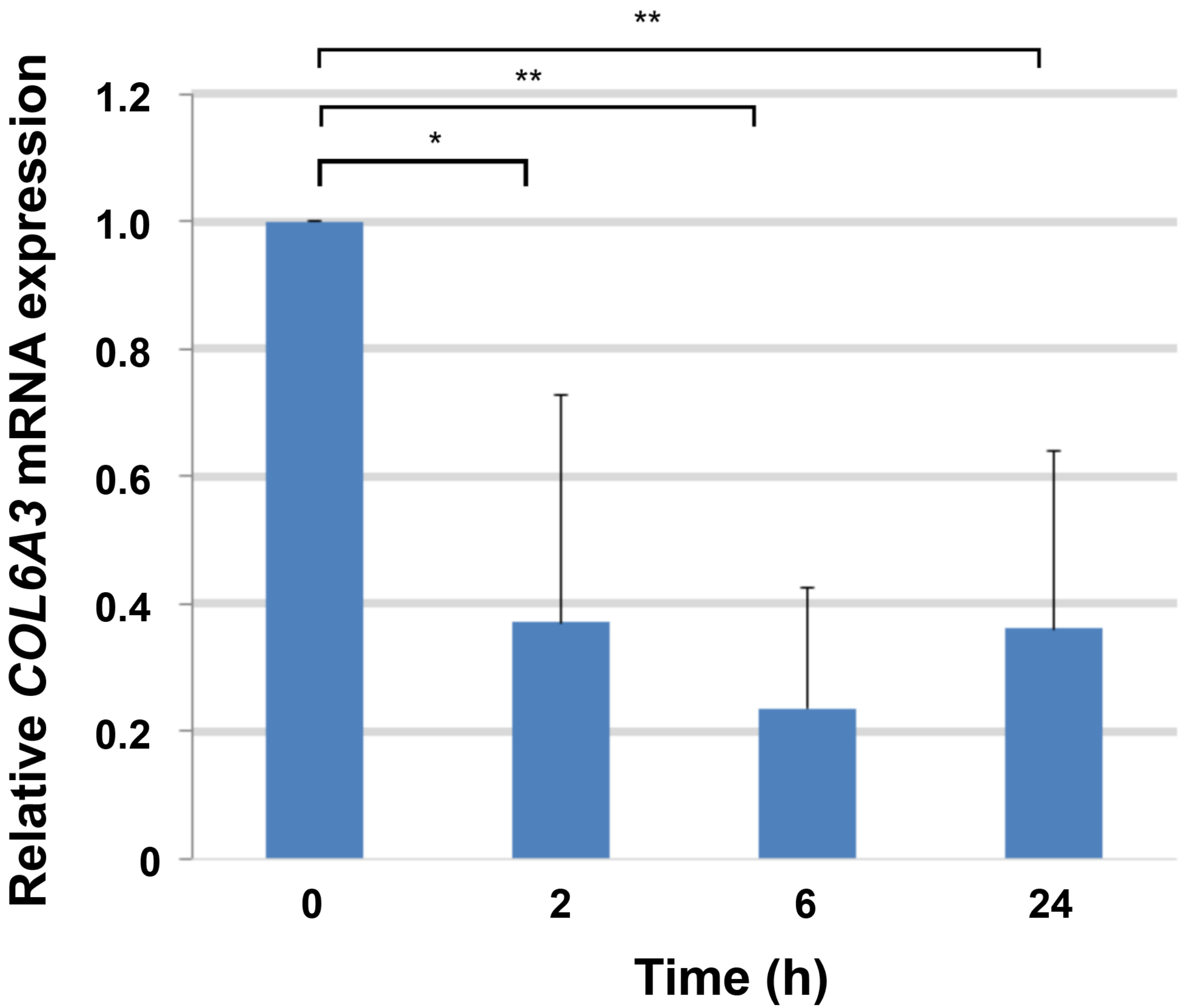

Supplementary Figure S3

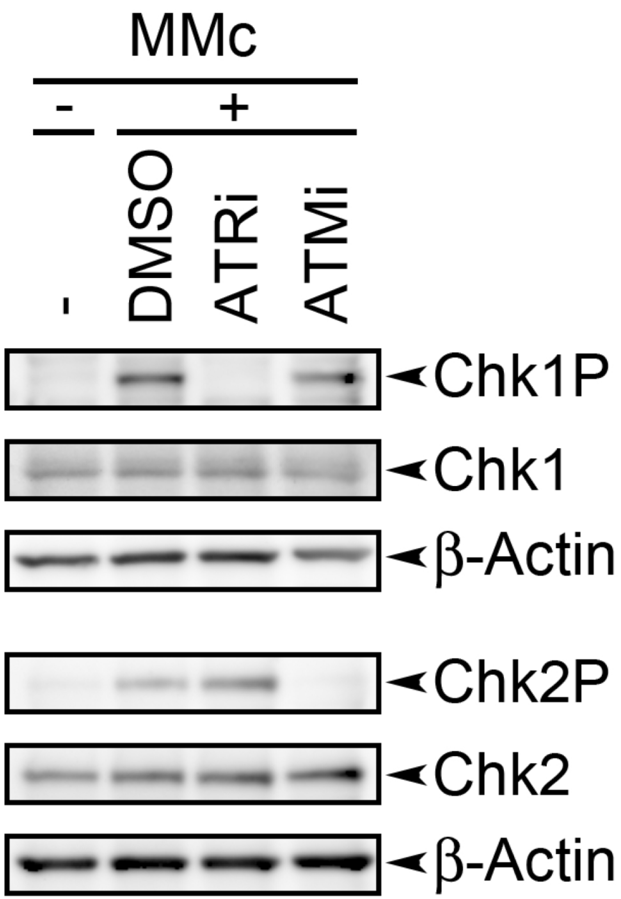

Supplementary Figure S4

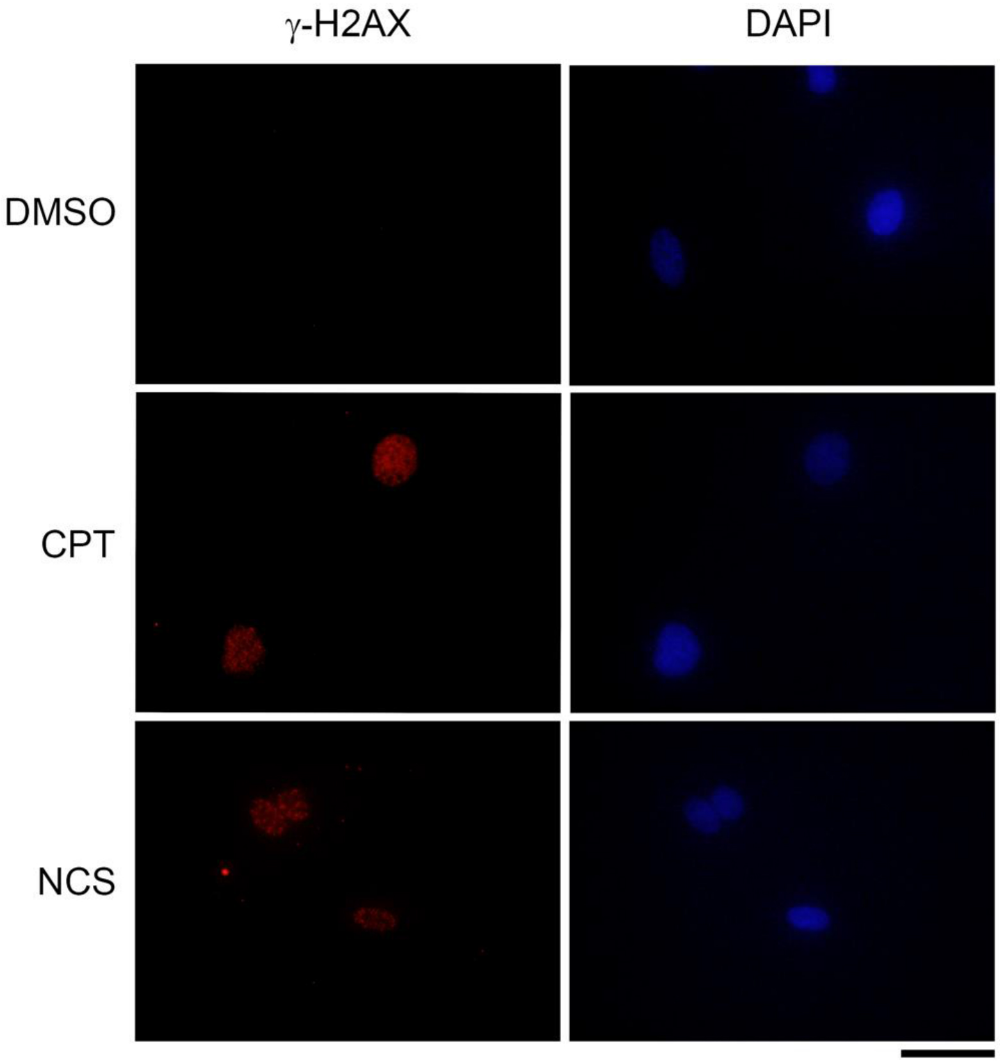

Supplementary Figure S5

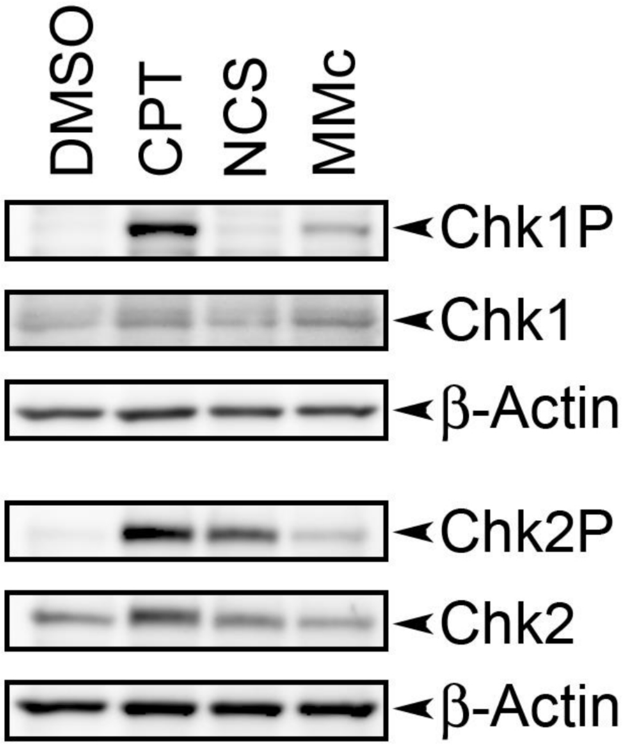

Supplementary Figure S6

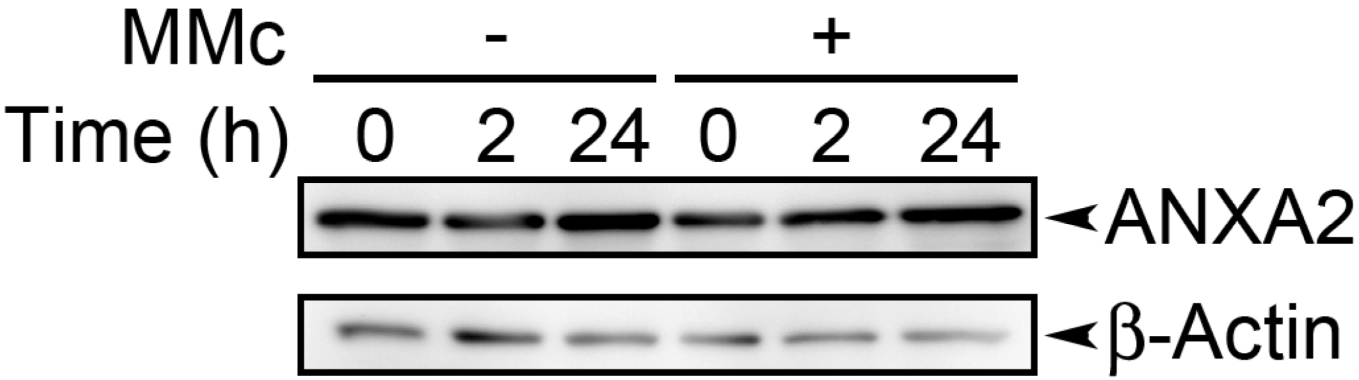

Supplementary Figure S7

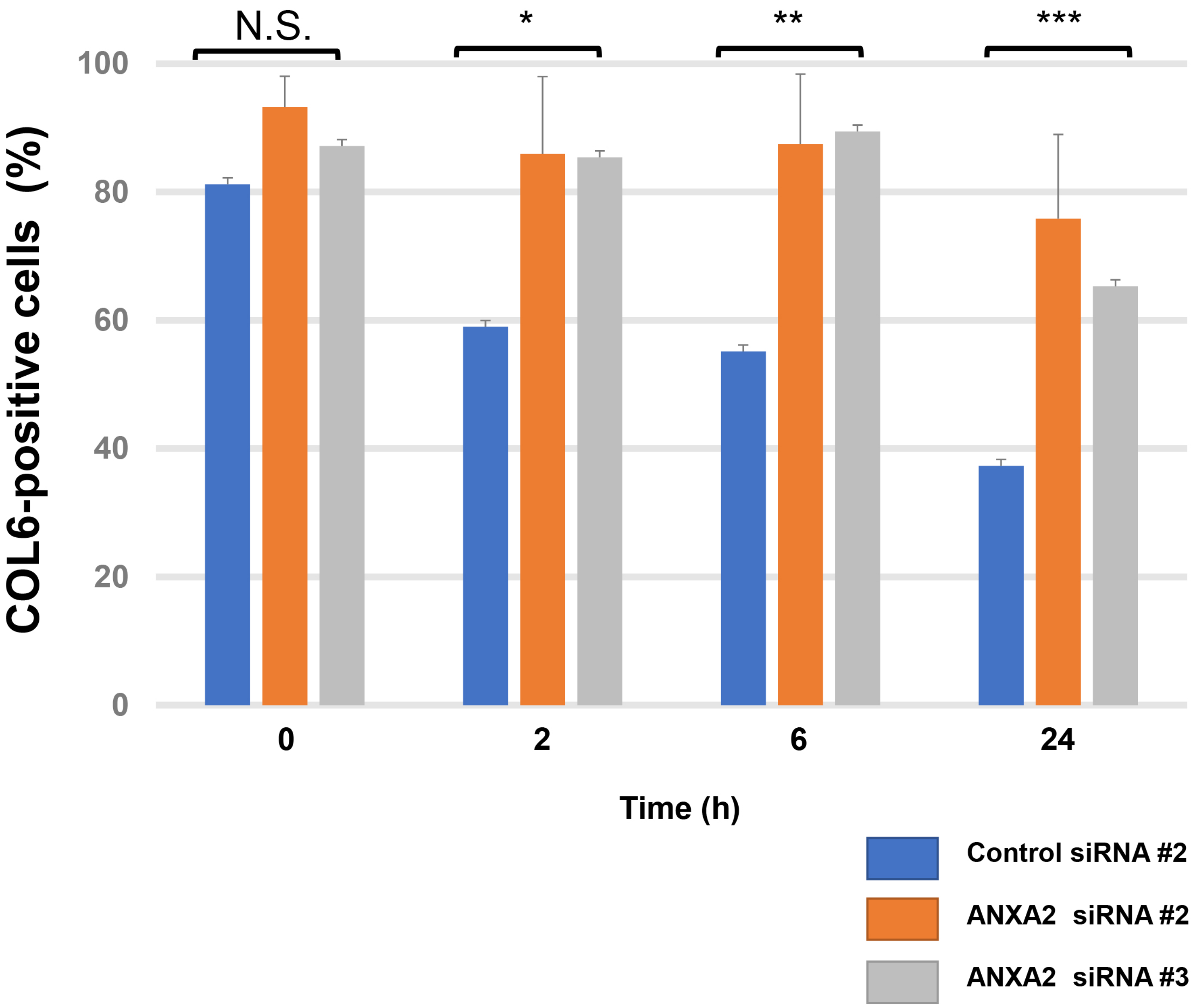

Supplementary Figure S8

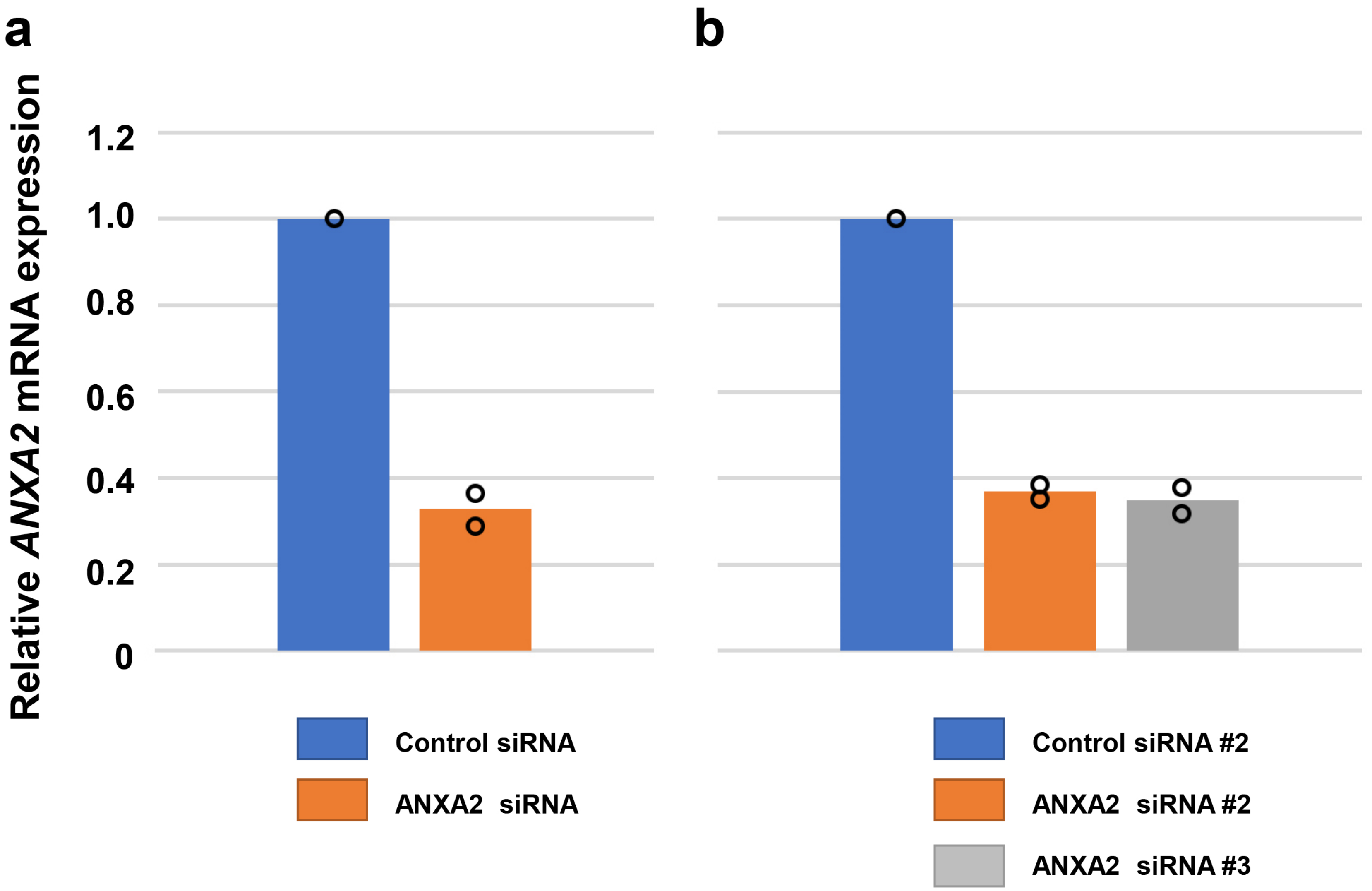

Supplementary Figure S9

a

Figure 2c

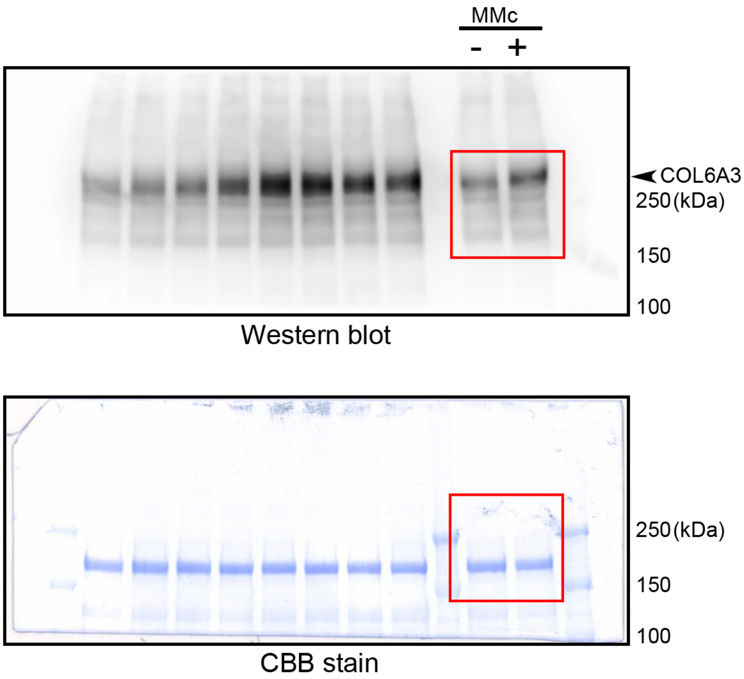

b

Figure 3a

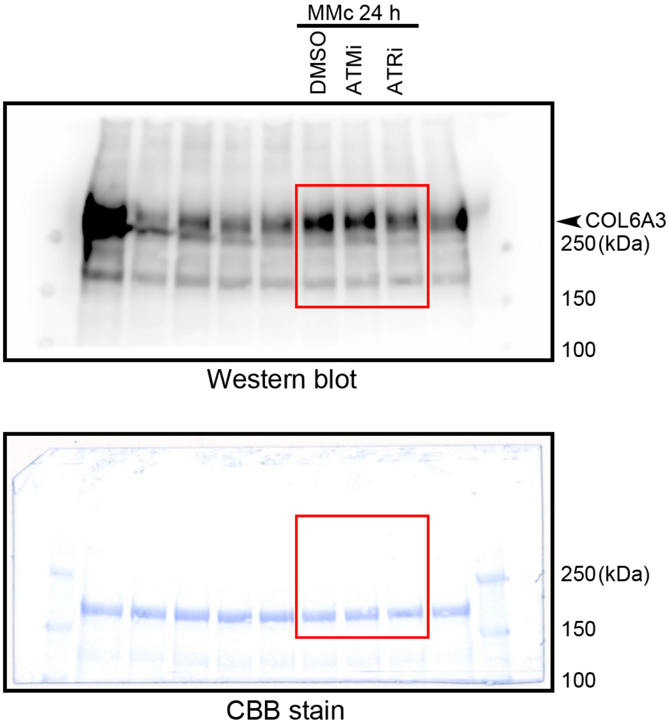

c

Figure 4a

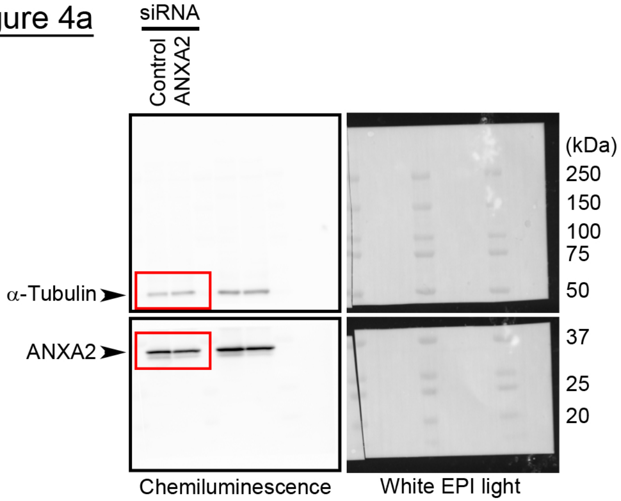

Supplementary Figure S9

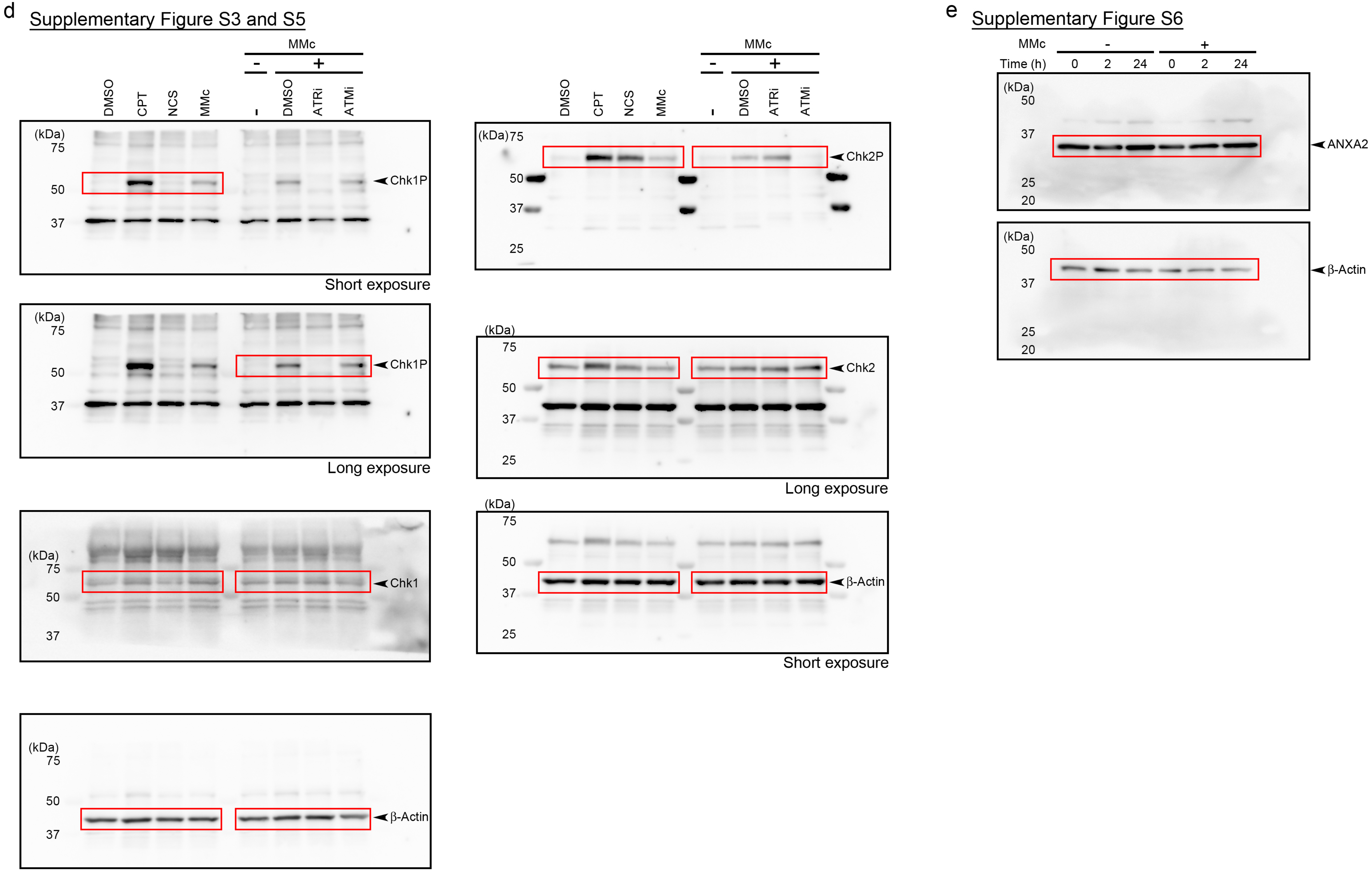

Supplementary Table. Definition and scores of pathological findings

	Pathological findings	Definition of scores
Glomerular lesions	Diffuse lesion (mesangial expansion)	0 normal or mild mesangial expansion,<br>1 mesangial expansion ≤ capillary lumen,<br>2 mesangial expansion = capillary lumen,<br>3 mesangial expansion ≥ capillary lumen
	Nodular lesion (nodular sclerosis)	0 (no nodular lesions),<br>1 (one or more lesions detected in all biopsy specimens regardless of nodular size)
	Subendothelial space widening (double contour of the basement membrane)	Double contour of the basement membrane (%) (in the peripheral capillary of the most severe glomerulus); 0 (<10%), 1 (10–25%), 2 (25–50%), 3 (≥50%)
	Exudative lesion	0 (not detected), 1 (detected for one or more lesions in all biopsy specimen)
	Mesangiolysis/microaneurysm	0 (not detected), 1 (detected for one or more lesions in all biopsy specimen)
	Peri-hilar neovascularization (polar vasculosis)	0 (not detected), 1 (detected for one or more lesions in all biopsy specimen)
	Global glomerulosclerosis/collapsing glomerulopathy ischemic nephropathy (%)	(Number of glomeruli with global glomerulosclerosis and collapsing glomerulopathy ischemic nephropathy)/total number of glomeruli (%)
	Segmental glomerulosclerosis (%)	Number of glomeruli with segmental glomerulosclerosis/number of all glomeruli (%)
	Glomerulomegaly	Glomeruli >250 μm in diameter , 0 (not detected), 1 (detected)
Interstitial lesions	Interstitial fibrosis and tubular atrophy (IFTA)	0 (no IFTA), 1 (<25%), 2 (25–50%), 3 (≥50%)
	Interstitial inflammation	0 (no cell infiltration), 1 (<25%), 2 (25–50%), 3 (≥50%)
Vascular lesions	Arteriolar hyalinosis	0 (no hyalinosis), 1 (one or more partial arteriolar hyalinosis), 2 (approximately 50% hyalinosis), 3 (more than 50% hyalinosis, or penetrating hyalinosis)
	Intimal thickening	0 (no intimal thickening), 1 (intimal thickness/media thickness <1), 2 (intimal thickening and intimal thickness/media thickness ≥1). Elastica van Gieson staining is helpful for assessments.

Supplementary Figure Legends

Supplementary Figure S1: Survival of HRGECs after the MMc treatment.
HRGECs treated with MMc (12 μg/mL, 2 h) were washed, and cultured without MMc for the indicated number of hours. Cells were stained with propidium iodide (PI) and Hoechst 33258 at the indicated time. Cells that had the PI signal and/or condensed chromatin were counted as dead cells and the percentage of dead cells was shown. A total of more than 100 cells of six independent fields per experiment was analyzed. Quantitative data are presented as the mean (n = 2).

Supplementary Figure S2: *COL6A3* mRNA expression of MMc-treated HRGECs.
HRGECs treated with MMc (12 μg/mL, 2 h) were washed, and cultured for the indicated number of hours without MMc. Total RNA was then extracted and 200 ng of total RNA were subjected to RT-PCR. Relative quantities of *COL6A3* mRNA in HRGECs were analyzed by the ΔΔCt method and compared to that for 0 h after MMc treatment. All data were normalized by the amount of *GAPDH* mRNA. Statistical significance was determined by Student's *t*-test. * $p < 0.05$, ** $p < 0.01$.

Supplementary Figure S3: Specific inhibitors of ATR or ATM suppress their kinase activities.
HRGECs were cultured without (−) or with DMSO, ATR inhibitor; VE-821 (ATRi) or ATM inhibitor; KU55933 (ATMi) for 1 h. Then MMc (12 μg/mL) was either added (+) or not (−), and cultured for 2 h. The cells were washed and cultured without or with DMSO, ATRi or ATMi for 2 h. Whole-cell extracts were analyzed by Western blotting using anti-phospho-Chk1/2 (Chk1P/2P), anti-Chk1/2 and anti-β-Actin antibodies. β-Actin was used as a loading control.

Supplementary Figure S4: CPT and NCS provoke the H2AX phosphorylation.
HRGECs treated with DMSO, CPT (1.0 μM, 4 h) or NCS (50 ng/mL, 4 h) were immunostained with anti-γ-H2AX antibody and counterstained with DAPI. Scale bar, 50 μm.

Supplementary Figure S5: ATM and ATR activation by CPT, NCS and MMc
HRGECs were treated with DMSO, CPT (1.0 μM, 4 h), NCS (50 ng/mL, 4 h) or MMc (12 μg/mL, 2 h). The cells were washed, and cultured without DNA damaging agents for 2 h. Whole-cell extracts were analyzed by Western blotting as described in Supplementary Fig. S3. β-Actin was used as a loading control.

Supplementary Figure S6: MMc does not affect the amount of ANXA2 protein.
HRGECs treated with (+) or without (−) MMc (12 μg/mL, 2 h) were washed and cultured for the indicated number of hours without MMc. Whole-cell extracts were subjected to Western blotting with anti-ANXA2 and anti-β-Actin antibodies. β-Actin was used as a loading control.

Supplementary Figure S7: ANXA2 is required for MMc-induced COL6 secretion.
The effect of ANXA2 knockdown on the MMc-induced COL6 secretion was confirmed by the use of different ANXA2 siRNA (#2 and #3). HRGECs transfected with Control siRNA #2, ANXA2 siRNA #2, or #3 were treated as shown in Fig. 4b, and immunostained with anti-COL6 antibody. The percentage of cells with cytoplasmic COL6 (COL6-positive cells) was scored as described in Fig. 4c, and numerical data are presented as mean ± SD (n

= 3 and the p value are (2 h, *$p < 0.05$; 6 h, **$p < 0.02$; 24 h, ***$p < 0.0001$ by the Kruskal-Wallis test). N.S., not significant.

Supplementary Figure S8: Reduction of *ANXA2* mRNA by ANXA2 siRNA transfection.
HRGECs were transfected with two different Control siRNA or three different ANXA2 siRNA. Forty-eight h post-transfection, total RNA (100 ng) was subjected to RT-PCR. Relative quantities of *ANXA2* mRNA in HRGECs were analyzed by the ΔΔCt method and compared to that for 0 h after MMc treatment. All data were normalized by the amount of *GAPDH* mRNA. (a) siRNAs used in Fig.4, (b) siRNAs used in Supplementary Fig. S7. Relative quantities are shown as the mean (n = 2).

Supplementary Figure S9 : Uncropped images from Western blot and CBB stain.
Uncropped Western blot images that correspond to Fig. 2c (a), Fig. 3a (b), Fig. 4a (c), Supplementary Fig. S3 (d), S5 (d), and S6 (e) are shown. (a and b) Uncropped scanning images of membranes after CBB stain are shown below their Western blot images.

Supplementary Methods

Cell survival assay

MMc-treated cells were stained with PI solution (421301, Biolegend, San Diego, CA) and Hoechst 33258 (Thermo Fisher). Cells that had both PI signal and/or condensed chromatin were counted as dead cells.

Reverse Transcription-PCR (RT-PCR)

Total RNA was isolated from HRGECs using the RNeasy Mini Kit (74134, Qiagen, Hilden, Germany). Total RNA (100 or 200 ng) was transcribed into cDNA by using SuperScript IV VILO Master Mix (Invitrogen) reverse transcriptase according to manufacturer instructions. Real-time PCR analyses were performed using TaqMan Gene Expression Assays (Applied Biosystems). The qRT-PCR reactions were set up in 96-well plates, in a final volume of 20 μL containing up to 4 μL of pre-amplified cDNA diluted 1:50, 1 μL of a pair of primers and a TaqMan probe, 5 μL of molecular water, and 10 μL of the Master Mix. The specific probes were *COL6A3* (Hs03929097) and *Glyceraldehyde-3-phosphate dehydrogenase* (*GAPDH*) (Hs00915125). We used the ABI Prism 7900HT Sequence Detection System (Applied Biosystems). The gene expression levels were normalized relative to those of *GAPDH* mRNA in the same samples using the ΔΔCt method.

Antibodies

The primary antibodies used in Supplementary experiments were as follows: anti-Chk1 pSer345 (2348, Cell Signaling Technology, Danvers, MA), anti-Chk1 (sc-8408, Santa Cruz Biotechnology), anti-Chk2 pThr68 (2197, Cell Signaling Technology), anti-Chk2 (3440, Cell Signaling Technology), anti-ANXA2 (sc-28385, Santa Cruz Biotechnology) and anti-β-Actin (sc-47778, Santa Cruz Biotechnology) antibodies for Western blotting and anti-γ-H2AX (05-636, Merck Millipore) antibody for Immunofluorescence.

siRNA treatment

Cells were transfected with siRNA by Lipofectamine RNAiMAX and incubated for 48 h as described in the manufacturer’s protocol. siRNAs used in Supplementary experiments were as follows: siRNAs specifically targeting human ANXA2 mRNA (#2, ANXA2HSS179173 and #3, ANXA2HSS179174, Invitrogen) or Control siRNA (#2, Stealth RNAi siRNA negative control (Invitrogen)).

Statistical analyses

Statistical analyses were performed using Student's *t*-test in Supplementary Fig. S2 and Kruskal-Wallis test in Supplementary Fig. S7. The differences were considered significant for values of $p < 0.05$.
